# Lymphocyte percentage as a valuable predictor of prognosis in lung cancer

**DOI:** 10.1111/jcmm.17214

**Published:** 2022-02-05

**Authors:** Hong Huang, Lei Li, Wenxin Luo, Yongfeng Yang, Yinyun Ni, Tingting Song, Yihan Zhu, Ying Yang, Li Zhang

**Affiliations:** ^1^ Institute of Clinical Pathology Key Laboratory of Transplantation Engineering and Immunology Ministry of Health West China Hospital Sichuan University Chengdu China; ^2^ Department of Pulmonary and Critical Care Medicine West China Hospital Sichuan University Chengdu China; ^3^ Precision Medicine Center West China Hospital Sichuan University Chengdu China

**Keywords:** clinical benefit, lung cancer, lymphocyte percentage, neutrophil percentage, poor prognosis, survival status

## Abstract

Lymphocytes and neutrophils are involved in the immune response against cancer. This study aimed to investigate the relationship between lymphocyte percentage/neutrophil percentage and the clinical characteristics of lung cancer patients, and to explore whether they could act as valuable predictors to ameliorate lung cancer prognosis. A total of 1312 patients were eligible to be recruited. Lymphocyte percentage and neutrophil percentage were classified based on their reference ranges. Survival curves were determined using Kaplan–Meier method, and univariate and multivariate cox regression analyses were performed to identify the significant predictors. Decision curve analysis was used to evaluate the clinical benefit. The results of both training and validation cohorts indicated that lymphocyte percentage exhibited high correlation with clinical characteristics and metastasis of lung cancer patients. Both lymphocyte percentage and neutrophil percentage were closely associated with survival status (all *p* < 0.0001). Low lymphocyte percentage could act as an indicator of poor prognosis; it offered a higher clinical benefit when combined with the clinical characteristic model. Our findings suggested that pretreatment lymphocyte percentage served as a reliable predictor of lung cancer prognosis, and it was also an accurate response indicator in lung adenocarcinoma and advanced lung cancer. Measurement of lymphocyte percentage improved the clinical utility of patient characteristics in predicting mortality of lung cancer patients.

**Main topics**

1. Pretreatment lymphocyte percentage was highly correlated with the clinical characteristics and metastasis of lung cancer patients.
2. Both lymphocyte percentage and neutrophil percentage were associated with the overall survival of lung cancer patients.
3. Lymphocyte percentage could serve as a powerful predictor of lung cancer prognosis, and it was also an accurate response indicator in lung adenocarcinoma and advanced lung cancer.
4. The integration of lymphocyte percentage improved the clinical utility of patient characteristic model in predicting mortality of lung cancer.

## 1 INTRODUCTION

Lung cancer accounts for 13% of new cancer cases and 26% of cancer deaths worldwide, with a five‐year relative survival rate of 18%. Ulike the steady rise in survival rates of most cancers, that of lung cancer has progressed more slowly.^1^, ^2^ Although traditional treatments can improve the prognosis of patients with advanced lung cancer, they are not specifically targeted and have contributed to little improvement in the outcomes of the disease.^3^, ^4^ Low‐dose computed tomography (LDCT) screening has the potential of diagnosing lung cancer at an early stage, which has demonstrated survival benefits by reducing existing mortality by up to 20%.^2^, ^5^, ^6^ However, the main limitation of LDCT screening is generation of false‐positive results, since it is unclear whether all lesions detected in asymptomatic participants will develop significant symptoms, and affect long‐term outcomes, suggesting that LDCT may be potentially harmful in large‐scale screening programmes.^7^, ^8^ Thus, more effective and low‐cost strategies need to be developed to consider patient acceptability, and assess the prognosis of lung cancer patients.

Circulating biomarkers in plasma and serum, which usually appear prior to imaging changes, can serve as indicators of tumour progression and predictors of prognosis.^9^, ^10^ Identifying reliable markers to better select patients for currently available and upcoming approaches, such as immunotherapy, will greatly assist clinical decision‐making. A variety of biomarkers, including carcinoembryonic antigen (CEA), cytokeratin 19 fragments (CYFRA 21–1), carbohydrate antigen (CA)125, CA199 and lactate dehydrogenase (LDH), have been identified to be associated with lung cancer prognosis.^11^, ^12^, ^13^, ^14^, ^15^

Inflammation is one of the hallmarks of cancer and plays a pivotal role in the modulation of tumour microenvironment. It can also highly influence tumorigenesis and tumour progression.^16^, ^17^ Different cells are known to be involved in the immune response against cancer, making the process dynamic and balanced.^18^ Lymphocytes and neutrophils are easy to measure and may provide a more convenient strategy for the study of cancer‐related inflammation. Neutrophil‐to‐lymphocyte ratio (NLR) has been evaluated in a variety of cancers, but its prognostic role remains controversial, which may explain the reason why it has not been incorporated into clinical practice.^19^, ^20^, ^21^, ^22^ For other haematological parameters, previous study has demonstrated that preoperative lymphocyte count is associated with node‐negative non‐small‐cell lung cancer (NSCLC) prognosis.^23^ An elevated neutrophil count has been shown to be a predictor of poor survival in metastatic melanoma.^24^ Overall changes in lymphocytes and neutrophils with regard to inflammation and the immune state may be expressed as lymphocyte percentage (LY%) and neutrophil percentage (NEUT%). Peripheral LY% reflects leukocytosis more directly than NLR does, the relative decrease of lymphocytes results in the diminishment of the immune response and increases the risk of cancer, and it has been reported to predict the survival more accurately than peripheral lymphocyte count in colorectal cancer.^25^ However, it was less considered in previous studies regarding the prognostic values of LY% and NEUT% in large cohort of patients with lung cancer.

In this study, a retrospective analysis was performed to investigate the relationship between LY%/NEUT% and clinical characteristics of lung cancer patients, and to evaluate whether LY% and NEUT% could be used for improving prediction of patient outcome.

## 2 MATERIALS AND METHODS

### 2.1 Ethics statement

This study was approved by the Medical Ethics Committee and Institutional Review Board of West China Hospital. Informed consent was obtained from all patients before study. All methods used in this study were performed following the approved protocols.

### 2.2 Patients

**FIGURE 1:**
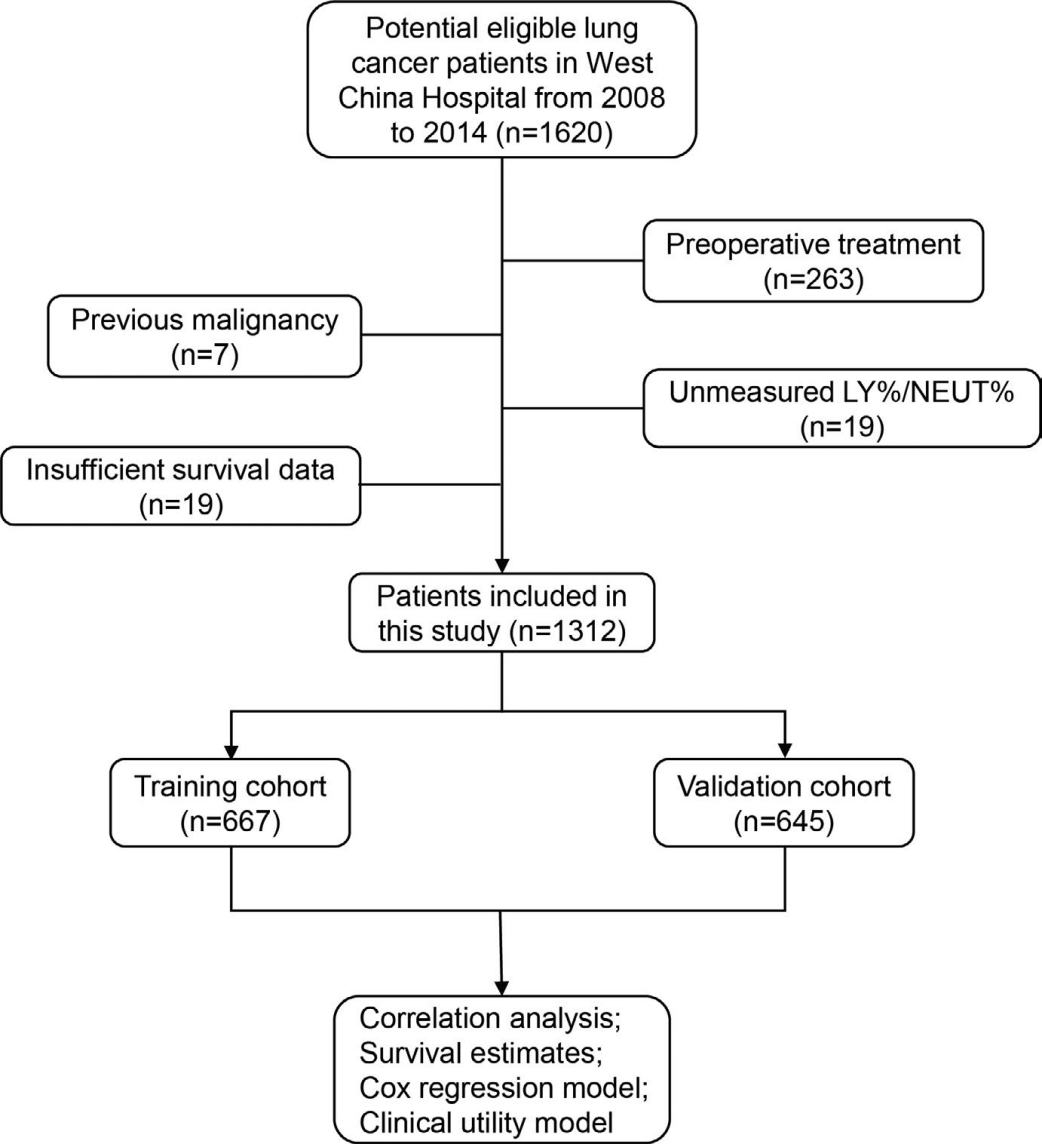
Flow chart of study population. LY%: lymphocyte percentage; NEUT%: neutrophil percentage

This study included 1312 patients diagnosed with lung cancer in West China Hospital from 2008 to 2014. On account of the differences in the levels of haematological indicators, 270 patients were excluded in advance due to preoperative treatment or history of cancer, while 38 patients were excluded owing to insufficient data of survival, or information regarding LY% or NEUT% in peripheral blood was not available (Figure 1). All clinical information was extracted from the medical records after lung cancer confirmed by biopsy. Information regarding metastasis was obtained using whole‐body CT scan, bone scan, lymph node biopsy and fibreoptic bronchoscopy. Survival status was determined on the last follow‐up day, and the overall survival time was defined as the length of time between the lung cancer confirmation date and the date of death or last follow‐up, which was done by visits or telephone inquiries.

### 2.3 Data collection and definition

Patient demographics, including age, gender, histological subtype, stage, smoking status and differentiation, were collected from medical records and pathology reports. Histological classification was determined according to the WHO guidelines,^26^ and lung cancer stage was defined based on the TNM Classification of Malignant Tumors (7th Edition).^27^ Data for complete blood counts including white blood cell count, neutrophil and lymphocyte counts were determined using the XE‐2100 and XE‐5000 systems (Sysmex, Kobe, Japan), and prior to any treatment such as surgery, radiotherapy and chemotherapy. LY% was defined as the percentage of lymphocyte count to white blood cell count, and NEUT% was defined as the percentage of neutrophil count to white blood cell count. In several analyses of cancer prognosis, the cut‐off value of LY% was defined as 20%,^28^, ^29^ and its normal level was considered to be 20%–50% in a study of lung cancer,^30^ which was in line with the clinical criteria of West China Hospital, and based on the diagnostic criteria and clinical experience, the reference range of NEUT% was defined as 40%–75%.

### 2.4 Statistical analysis

Continuous variables were presented as median (range). Categorical variables were presented as percentage (%). Chi‐square test was performed to determine the statistical significance of categorical data. For survival analysis, the log‐rank test was used for univariate and multivariate cox regression analysis. To measure effects of variables on survival, statistical significance was expressed as hazard ratio (HR), at 95% confidence interval (CI). Survival curves were constructed using the Kaplan–Meier method. With regard to clinical utility, the net benefit was measured by decision curve analysis (DCA) according to Vickers et al.^31^ *p* < 0.05 was considered statistically significant. Statistical analyses were performed using SPSS v23.0 (IBM Inc., Chicago, USA) and R software v3.6.1. Figures were completed using GraphPad Prism 8.0 (GraphPad Software Inc., La Jolla, USA).

## 3 RESULTS

### 3.1 Patient Characteristics

A total of 1312 lung cancer patients were randomly divided into two cohorts (Table S1), with 667 cases in training cohort and the remaining 645 cases in validation cohort, of which 894 were male (68.1%) and 418 were female (31.9%), with a median age of 60 years (range, 16–93 years). The dominant histological subtypes included adenocarcinoma (ADC, 675 cases, 51.4%), squamous carcinoma (SCC, 338 cases, 25.8%) and small‐cell lung cancer (SCLC, 213 cases, 16.2%), while the remaining 86 patients included large cell lung cancer and adenosquamous carcinoma. About half of the participants (667 cases) were stage IV lung cancer patients, the majority of the subjects were current or ex‐smokers (732 cases, 55.8%), and 894 cases (68.1%) had metastases at different sites. Additionally, the median overall survival time was 18 months, with a follow‐up time of five years. Among the participants, 585 (44.6%) had lymphocyte percentage below 20%, while 367 (28.0%) had neutrophil percentage above 75%.

### 3.2 Correlation of LY% and NEUT% with clinical characteristics in all lung cancer patients

The association between LY% and the clinicopathologic characteristics of lung cancer patients was investigated (Table 1). In the training cohort, 365 cases were identified to be within the reference range, while 302 cases were below 20%. For the validation cohort, there are 362 and 283 cases, respectively. The results indicated that low LY% was correlated with male (both cohorts, *p* = 0.013), stage IV (T: *p* = 0.001, V: *p* = 0.000) and undifferentiated patients (T: *p* = 0.001, V: *p* = 0.000). Additionally, LY% was associated with histological subtypes (T: *p* = 0.002, V: *p* = 0.034) and organ metastasis: bone (T: *p* = 0.002, V: *p* = 0.029), liver (T: *p* = 0.045, V: *p* = 0.005) and pleura (T: *p* = 0.000, V: *p* = 0.037). No significant differences were observed in age, smoking status and metastasis to other sites.

**TABLE 1:** Analysis of LY% in all lung cancer patients

Training cohort	No. (%)		Total (n=667)	*p* value
	20–50 (n=365)	<20 (n=302)		
Basic characteristics				
Age				0.002**
<45	32(8.7)	17(5.6)	49	
45–60	174(47.7)	112(37.1)	286	
>60	159(43.6)	173(57.3)	332	
Sex				0.013*
Male	229(62.7)	217(71.9)	446	
Female	136(37.3)	85(28.1)	221	
Histological subtype				0.002**
SCC	75(20.5)	94(31.1)	169	
ADC	187(51.2)	151(50.0)	338	
SCLC	77(21.1)	42(13.9)	119	
Others	26(7.2)	15(5.0)	41	
Stage				0.001**
I	43(1.8)	14(4.7)	57	
II	34(9.3)	17(5.6)	51	
III	86(23.6)	84(27.8)	170	
IV	178(48.8)	168(55.6)	346	
Unknown	24(6.5)	19(6.3)	43	
Smoking status				0.093
Never smoking	176(48.2)	126(41.7)	302	
Current or ex‐smoker	189(51.8)	176(58.3)	365	
Differentiation				0.001**
Undifferentiated	234(64.1)	215(71.2)	449	
Poor	43(11.8)	48(15.9)	91	
Moderate	79(21.6)	32(10.6)	111	
Well	3(0.8)	1(0.3)	4	
Unknown	6(1.7)	6(2.0)	12	
Metastasis				
Brain				0.976
No	329(90.1)	272(90.1)	601	
Yes	36(9.9)	30(9.9)	66	
Bone				0.002**
No	314(86)	232(76.8)	546	
Yes	51(14.0)	70(23.2)	121	
Liver				0.045*
No	341(93.4)	269(89.1)	610	
Yes	24(6.6)	33(10.9)	57	
Adrenal gland				0.736
No	342(93.7)	281(93.0)	623	
Yes	23(6.3)	21(7.0)	44	
Lymph node				0.180
No	170(46.6)	125(41.4)	295	
Yes	195(53.4)	177(58.6)	372	
Intrapulmonary				0.244
No	324(88.8)	259(85.8)	583	
Yes	41(11.2)	43(14.2)	84	
Pleural				0.000***
No	333(91.2)	248(82.1)	581	
Yes	32(8.8)	54(17.9)	86	
Mediastinal				0.514
No	356(97.5)	292(96.7)	648	
Yes	9(2.5)	10(3.3)	19	

Validation cohort	No. (%)		Total (n=645)	*p* value
	20–50 (n=362)	<20 (n=283)		
Basic characteristics				
Age				0.826
<45	28(7.8)	24(8.5)	52	
45–60	167(46.1)	124(43.8)	291	
>60	167(46.1)	135(47.7)	302	
Sex				0.013*
Male	237(65.5)	211(74.6)	448	
Female	125(34.5)	72(25.4)	197	
Histological subtype				0.034*
SCC	84(23.2)	87(30.7)	171	
ADC	205(56.6)	132(46.6)	337	
SCLC	50(13.8)	44(15.5)	94	
Others	23(6.4)	20(7.2)	43	
Stage				0.000***
I	42(11.6)	13(4.6)	55	
II	40(11.1)	19(6.7)	59	
III	89(24.6)	71(25.1)	160	
IV	159(43.9)	162(57.2)	321	
Unknown	32(8.8)	18(6.4)	50	
Smoking status				0.338
Never smoking	162(44.8)	116(41.0)	278	
Current or ex‐smoker	200(55.2)	167(59.0)	367	
Differentiation				0.000***
Undifferentiated	229(63.3)	205(72.4)	434	
Poor	43(11.9)	49(17.3)	92	
Moderate	87(24.0)	26(9.2)	113	
Well	2(0.5)	2(0.7)	4	
Unknown	1(0.3)	1(0.4)	2	
Metastasis				
Brain				0.465
No	331(91.4)	254(89.8)	585	
Yes	31(8.6)	29(10.2)	60	
Bone				0.029*
No	308(85.1)	222(78.4)	530	
Yes	54(14.9)	61(21.6)	115	
Liver				0.005**
No	342(94.5)	250(88.3)	592	
Yes	20(5.5)	33(11.7)	53	
Adrenal gland				0.002**
No	357(98.6)	267(94.3)	624	
Yes	5(1.4)	16(5.7)	21	
Lymph node				0.020*
No	170(47.0)	107(37.8)	277	
Yes	192(53.0)	176(62.2)	368	
Intrapulmonary				0.198
No	326(90.1)	263(92.9)	589	
Yes	36(9.9)	20(7.1)	56	
Pleural				0.037*
No	324(89.5)	239(84.5)	563	
Yes	38(10.5)	44(15.5)	82	
Mediastinal				0.063
No	358(98.9)	274(96.8)	632	
Yes	4(1.1)	9(3.2)	13	

* *p *< 0.05, **p < 0.01, ***p < 0.001. LY%: lymphocyte percentage; SCC: lung squamous carcinoma; ADC: lung adenocarcinoma; SCLC: small‐cell lung cancer; poor: poorly differentiated; moderate: moderately differentiated; well: well differentiated.

Analysis of NEUT% showed that, in the training and validation cohorts, 183 and 184 cases, respectively, were beyond the reference range. Distinct differences in histological classification (T: *p* = 0.000, V: *p* = 0.003) and differentiation (T: *p* = 0.001, V: *p* = 0.020) were detected, and NEUT% showed a relatively poor correlation with metastasis in all lung cancer patients (Table S2).

### 3.3 Correlation of LY% and NEUT% with clinical characteristics in different histological subtypes

For the purpose of treatment, lung cancer was classified as SCLC and NSCLC, ADC and SCC accounted for more than 80% of NSCLC cases.^32^, ^33^ Hence, classification analyses of LY% and NEUT% in ADC, SCC and SCLC were performed.

A further analysis of 675 ADC cases for LY% classification included 392 normal and 283 aberrant cases (Table 2), indicating that decreased LY% was closely correlated with male ADC patients (*p* = 0.006). It was also found that LY% was normal in early stage lung cancer but abnormal in advanced stage (*p* = 0.000). Additionally, the LY% of undifferentiated and poorly differentiated ADC patients were lower compared to those with moderate differentiation (*p* = 0.000). For well‐differentiated ADC patients, cases were too few to draw a conclusion (5 cases in total). Besides, LY% showed a strong correlation with occurrence of metastasis in ADC patients. It was associated with bone (*p* = 0.000), pleural (*p* = 0.000), adrenal gland (*p* = 0.008), liver (*p* = 0.007), lymph node (*p* = 0.025) and mediastinal (*p* = 0.045) metastasis. 338 SCC cases were analysed, and what made the analysis different from the others was that the number of cases in abnormal LY% group (179 cases) was higher than normal LY% group (159 cases). The results indicated that patients older than 60 years had relatively low LY% levels (*p* = 0.043). In addition, the results of differentiation were consistent with those of ADC analysis of LY% (*p* = 0.017), while metastasis analysis showed no significant differences. Among the 213 patients diagnosed with SCLC, 86 had abnormal LY%. It was found that low LY% was correlated with the adverse characteristic of advanced stage (IV) (*p* = 0.048) and was associated with liver (*p* = 0.032), lymph node (*p* = 0.014) and pleural (*p* = 0.014) metastatic lesions (Table S3).

**TABLE 2:** Analysis of LY% in ADC patients

LY%	No. (%)		Total (n = 675)	*p* value
	20–50 (n = 392)	<20 (n = 283)		
Basic characteristics				
Age				0.639
<45	38(9.7)	29(10.2)	67	
45–60	168(42.9)	111(39.2)	279	
>60	186(47.4)	143(50.5)	329	
Sex				0.006**
Male	192(49.0)	169(59.7)	361	
Female	200(51.0)	114(40.3)	314	
Stage				0.000***
I	57(14.5)	10(3.6)	67	
II	38(9.8)	13(4.6)	51	
III	68(17.3)	55(19.4)	123	
IV	210(53.6)	190(67.1)	400	
Unknown	19(4.8)	15(5.3)	34	
Smoking status				0.434
Never smoking	243(62.0)	167(59.0)	410	
Current or ex‐smoker	149(38.0)	116(41.0)	265	
Differentiation				0.000***
Undifferentiated	235(59.9)	210(74.2)	445	
Poor	33(8.4)	41(14.5)	74	
Moderate	118(30.2)	28(9.8)	146	
Well	4(1.0)	1(0.4)	5	
Unknown	2(0.5)	3(1.1)	5	
Metastasis				
Brain				0.135
No	349(89.0)	241(85.2)	590	
Yes	43(11.0)	42(14.8)	85	
Bone				0.000***
No	321(81.9)	198(70.0)	519	
Yes	71(18.1)	85(30.0)	156	
Liver				0.007**
No	371(94.6)	252(89.0)	623	
Yes	21(5.4)	31(11.0)	52	
Adrenal gland				0.008**
No	382(97.4)	264(93.3)	646	
Yes	10(2.6)	19(6.7)	29	
Lymph node				0.025*
No	192(49.0)	114(40.3)	306	
Yes	200(51.0)	169(59.7)	369	
Intrapulmonary				0.959
No	343(87.5)	248(87.6)	591	
Yes	49(12.5)	35(12.4)	84	
Pleural				0.000***
No	340(86.7)	216(76.3)	556	
Yes	52(13.3)	67(23.7)	119	
Mediastinal				0.045*
No	387(98.7)	273(96.5)	660	
Yes	5(1.3)	10(3.5)	15	

* *p *< 0.05, **p < 0.01, ***p < 0.001. LY%: lymphocyte percentage; ADC: lung adenocarcinoma; poor: poorly differentiated; moderate: moderately differentiated; well: well differentiated.

For NEUT%, the results showed that it was correlated with the stage of ADC (*p* = 0.005) and SCLC (*p* = 0.039), and differentiation analysis revealed changes similar to those of LY% in ADC (*p* = 0.000) and SCC (*p* = 0.011). NEUT% had relevance regarding several metastatic sites, including liver (*p* = 0.016) and adrenal gland (*p* = 0.001) in ADC, bone (*p* = 0.010) in SCC, and liver (*p* = 0.016) in SCLC (Table S4).

### 3.4 LY% and NEUT% were associated with overall survival of lung cancer

**FIGURE 2:**
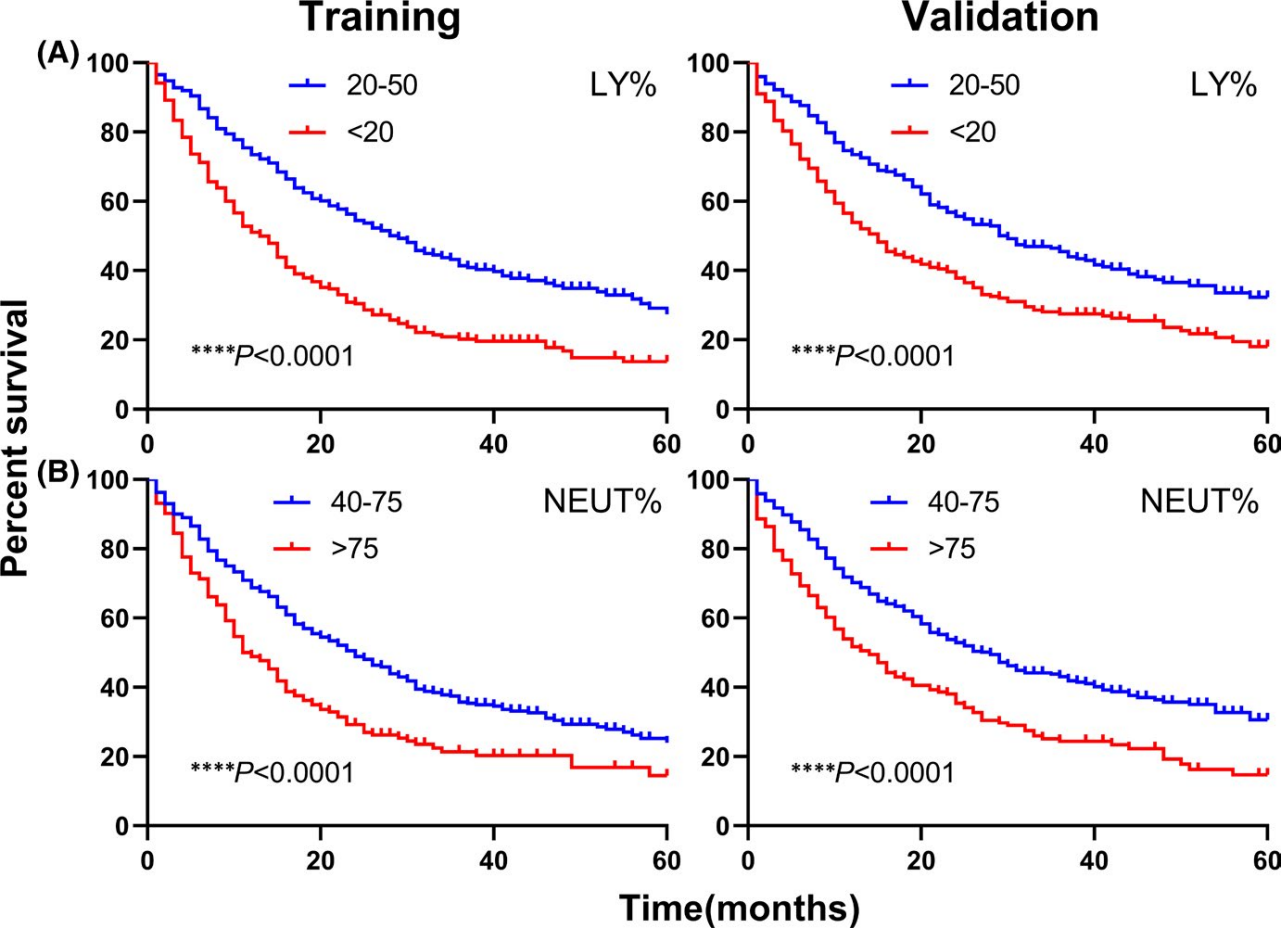
Kaplan–Meier curves for overall survival according to LY% (A) and NEUT% (B) in training and validation cohorts of all lung cancer patients. ****p < 0.0001. LY%: lymphocyte percentage; NEUT%: neutrophil percentage

The overall survival time of patients in training and validation cohorts was evaluated using Kaplan–Meier survival curves. As shown in Figure 2, low LY% was strongly correlated with poor survival status (both cohorts, *p* < 0.0001), and NEUT% showed worse overall survival in higher level (both cohorts, *p* < 0.0001).

**FIGURE 3:**
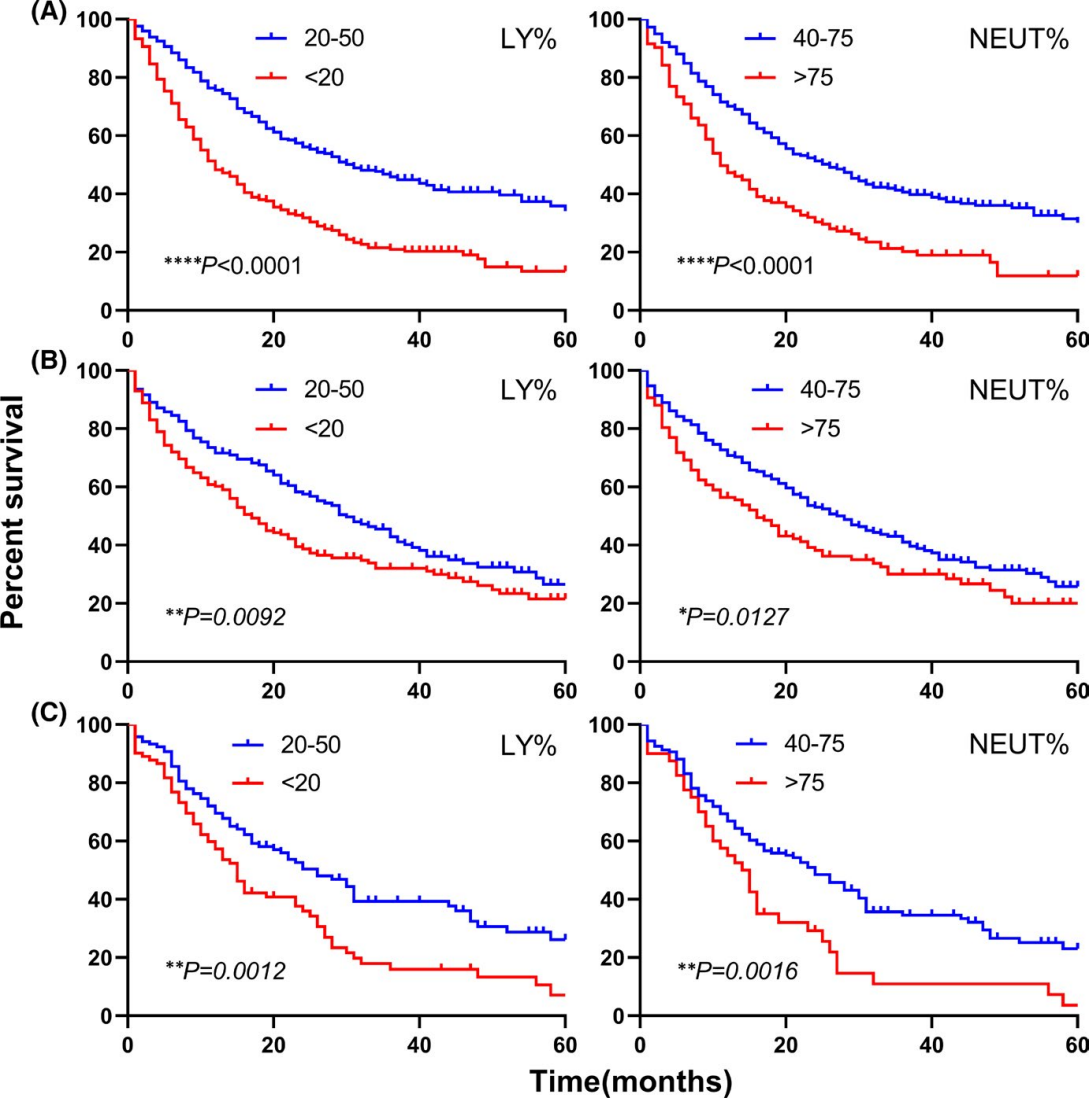
Kaplan–Meier curves for overall survival stratified by histological subtypes. Kaplan–Meier curves for overall survival according to LY% and NEUT% in ADC (A), SCC (B) and SCLC (C). *p < 0.05, **p < 0.01, ****p < 0.0001. LY%: lymphocyte percentage; NEUT%, neutrophil percentage; ADC, lung adenocarcinoma; SCC, lung squamous carcinoma; SCLC, small‐cell lung cancer

**FIGURE 4:**
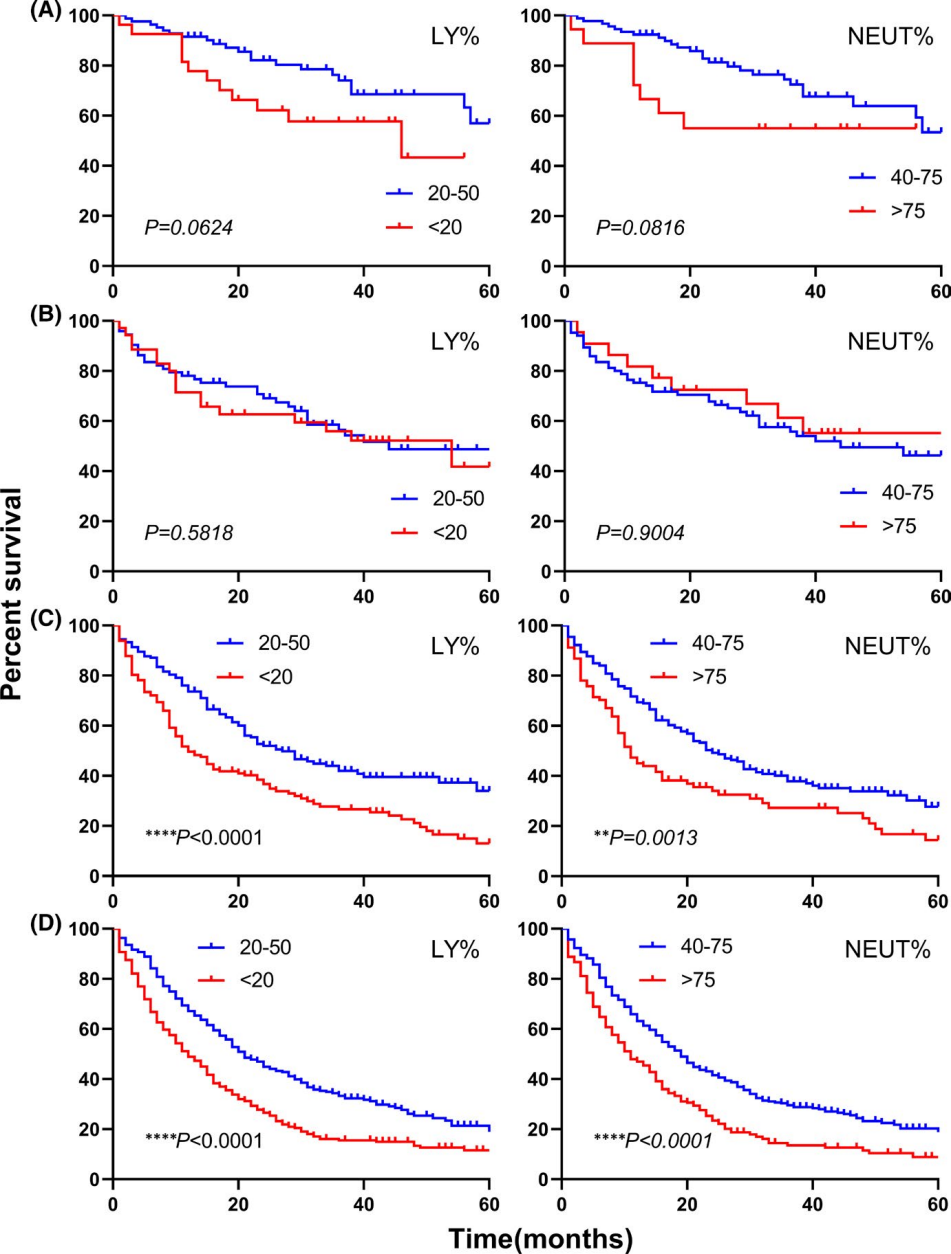
Kaplan–Meier curves for overall survival stratified by TNM stages. Kaplan–Meier curves for overall survival according to LY% and NEUT% in stage I (A), stage II (B), stage III (C), and stage IV (D). **p < 0.01, ****p < 0.0001. LY%, lymphocyte percentage; NEUT%, neutrophil percentage

After stratification based on histology, significant differences were found in each subtype (Figure 3). Patients with lower LY% exhibited worse prognosis in ADC (*p* < 0.0001), SCC (*p* = 0.0092) and SCLC (*p* = 0.0012), and higher NEUT% was more associated with ADC (*p* < 0.0001), SCC (*p* = 0.0127) and SCLC (*p* = 0.0016) mortalities. As for survival curves of TNM stages (Figure 4), aberrant LY% levels were associated with shorter overall survival in stage III and IV (both stages, *p* < 0.0001), while in early stages (I and II), no differences were observed (I: *p* = 0.0624, II: *p* = 0.5818). NEUT% results were in accordance with those of LY% (I: *p* = 0.0816, II: *p* = 0.9004, III: *p* = 0.0013, IV: *p* < 0.0001).

### 3.5 LY% could serve as a valuable predictor of prognosis in lung cancer

**FIGURE 5:**
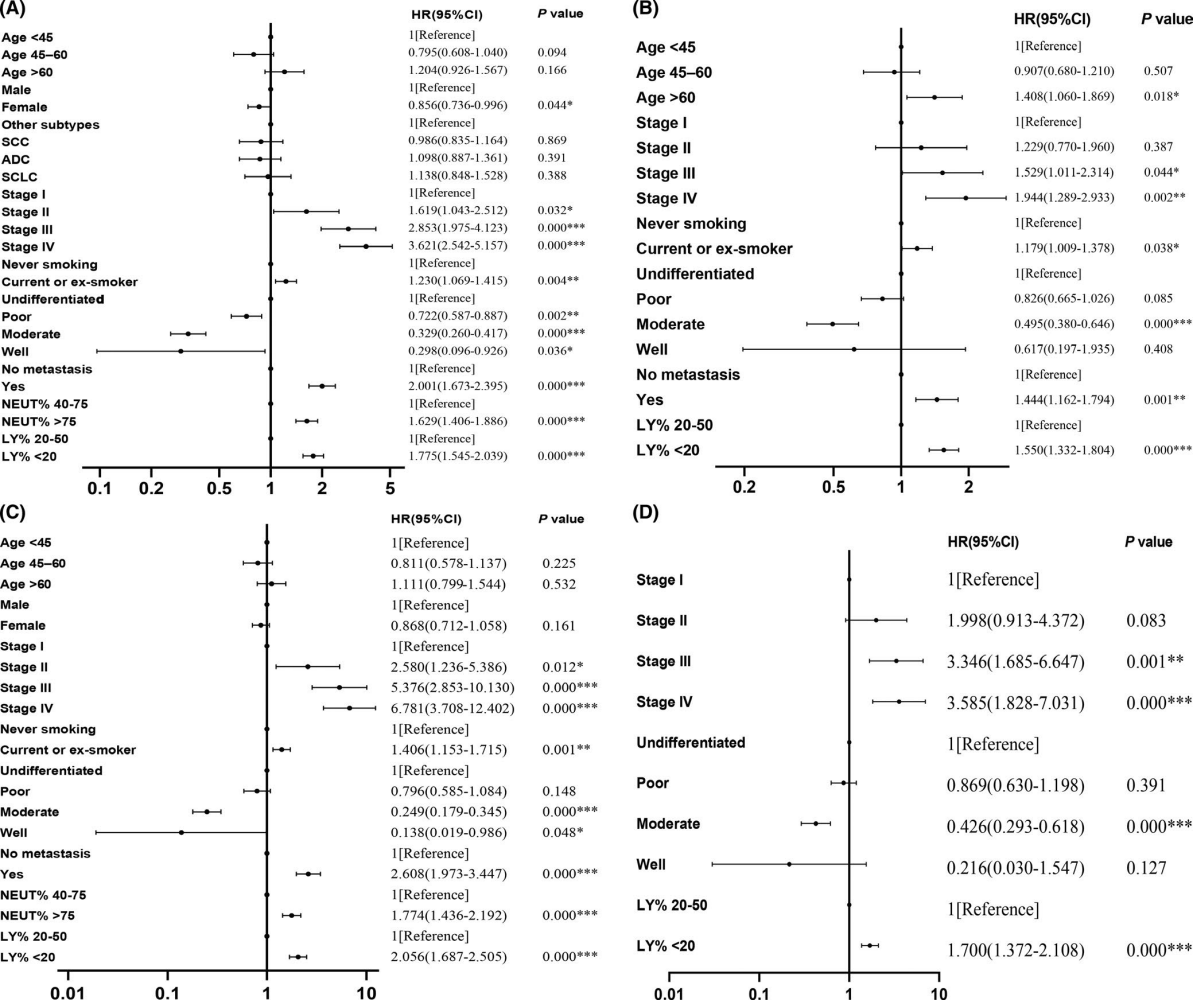
Forest plots showed the combined relation hazard ratio (HR) with 95% confidence interval (CI) for the association between patient characteristics and overall survival of lung cancer patients. (A) Univariate analysis of all patients. (B) Multivariate analysis of all patients. (C) Univariate analysis of ADC patients. (D) Multivariate analysis of ADC patients. *p < 0.05, **p < 0.01, ***p < 0.001. SCC: lung squamous carcinoma; ADC: lung adenocarcinoma; SCLC: small‐cell lung cancer; poor: poorly differentiated; moderate: moderately differentiated; well: well differentiated; NEUT%: neutrophil percentage; LY%: lymphocyte percentage

Univariate and multivariate cox regression models were introduced to measure prognostic predictors of lung cancer patients. The univariate analysis revealed that aberrant levels of LY% and NEUT% conferred unfavourable prognosis (both *p* = 0.000). Sex, stage, smoking status, differentiation and metastasis were associated with prognosis in all lung cancer patients (Figure 5A). A multivariate regression analysis was conducted for 8 variables with statistically significant differences (*p* < 0.1) in univariate analysis. The HR increased to 1.550 (95% CI: 1.332–1.804, *p* = 0.000) in low LY% group, compared with the reference, suggesting that low LY% could serve as an important predictor of poor prognosis for lung cancer patients. Moreover, age older than 60 years (*p* = 0.018), advanced stage (III: *p* = 0.044, IV: *p* = 0.002), smoking (*p* = 0.038) and metastasis occurrence (*p* = 0.001) were also correlated with poor prognosis, while moderate differentiation was associated with favourable prognosis of lung cancer (HR: 0.495, 95% CI: 0.380–0.646, *p* = 0.000). The well‐differentiated patients did not appear to be statistically significant. However, due to the limited number of such patients (eight cases), the results should be treated with caution (Figure 5B).

In addition, univariate and multivariate analyses in specific histological subtypes were performed. When variables identified as significant (*p* < 0.1) in univariate analysis were included in multivariate analysis as covariates, the results indicated that LY% remained an accurate predictor of prognosis in ADC (HR: 1.700, 95% CI: 1.372–2.108, *p* = 0.000). Advanced stages were significantly associated with poor prognosis of ADC (III: *p* = 0.001; IV: *p* = 0.000). Moderate differentiation (*p* = 0.000) was also closely correlated to prognosis (Figure 5C‐D). For SCC, LY% was also identified as a predictor of prognosis (*p* = 0.019), and regarding SCLC, a high NEUT% level conferred an unfavourable outcome (*p* = 0.027). Patients were then divided into subgroups according to specific stages. The HR of LY% increased to 1.698 (1.263–2.284, *p* = 0.000) in stage III and to 1.575 (1.303–1.903, *p* = 0.000) in stage IV, suggesting LY% could serve as a predictor of prognosis in advanced lung cancer (Table S5). Some additional analyses of current data had also been performed, and the results revealed that LY% was still correlated with survival outcome in patients with unfavourable characteristics (undifferentiated: *p* = 0.000; metastasis: *p* = 0.000). Further, in patients without metastasis, high NEUT% was a predictor of poor prognosis (*p* = 0.001, Table S5).

### 3.6 Clinical utility of LY% integration model in lung cancer prognosis

**FIGURE 6:**
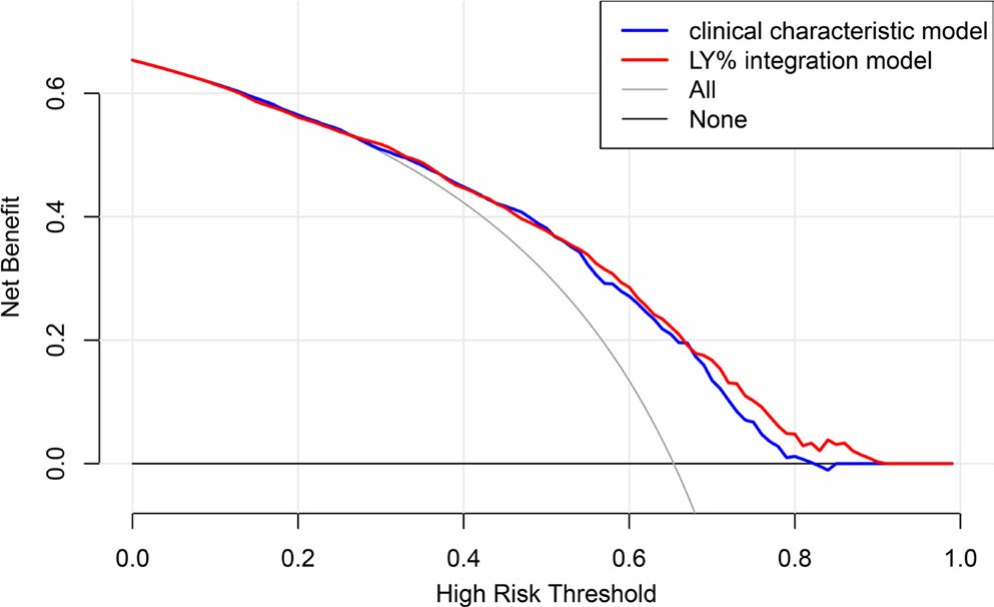
Decision curves for the clinical characteristic model and LY% integration model to predict the mortality of patients with lung cancer. Blue line: the net benefit of treating patients based on clinical characteristics (age, stage, smoking status, differentiation and metastasis); red line: the net benefit of treating patients according to LY% and clinical characteristics; grey line: net benefit of the treat all strategy; black line: net benefit of the treat none strategy. LY%, lymphocyte percentage

Decision curve analysis was performed to evaluate the clinical net benefit of the multivariate prognostic prediction model integrating LY% on overall survival of lung cancer patients. Patient characteristics associated with poor prognosis in multivariate regression analysis were included as clinical characteristic model (age, stage, smoking status, differentiation and metastasis), LY% integrated into clinical characteristic model was set as LY% integration model, so any difference between the two curves indicated the additional role of LY% (Figure 6). The analysis revealed that clinical characteristic model was useful across high‐risk threshold range of ~30%–80%. For low thresholds (<30%), the benefits of using the model were no different from the treat all strategy. For thresholds above this range (>80%), the model no longer provided meaningful distinctions from the treat none strategy. In addition, LY% integration model was useful between threshold probabilities of ~30%–90%. From thresholds of 30%–50%, the two curves intersected and depicted little difference in net benefit. However, the multivariate model that combined LY% with age, stage, smoking status, differentiation and metastasis provided a higher net benefit over a relatively high threshold range from 50% to 90%, compared to the model of clinical characteristics only. Consequently, the integration of LY% achieved better clinical benefit in lung cancer prognosis.

## 4 DISCUSSION

Research for the present large‐scale samples of lung cancer provided an opportunity to explore the prognostic value of peripheral cells from a different perspective. In order to measure the prognostic performance of clinical characteristics more comprehensively, patients of all types and stages were recruited. Characteristics associated with prognosis were incorporated into clinical utility model to evaluate clinical net benefit, which was applicable to all lung cancer patients. Our observation demonstrated that pretreatment LY% and NEUT% were significantly correlated with clinical parameters, including age, histological classification, stage, differentiation and occurrence of metastasis at different sites. Considering the percentage of peripheral circulating cells and individual survival status in the cohorts, LY% was highly associated with patient outcomes. The results suggested that low LY% was a more valuable predictor of poor prognosis, and it had an additional clinical benefit for patients with lung cancer.

Based on observations that tumours often arise at chronic inflammation sites, and that inflammatory cells are present in tumour biopsy samples, it is thus evident inflammatory cells play a crucial role in tumour development and progression.^17^, ^34^ In the early neoplastic process, inflammatory cells are powerful tumour promoters, which create a favourable environment for tumour growth, facilitating genomic instability and angiogenesis.^35^ Inflammation is also involved in various phases of tumorigenesis process, from initiation through promotion to metastasis. Inflammatory processes always accompany cancer, and persistent chronic inflammation in cancer tissue gives rise to inhibition of anti‐tumour immunity via a variety of mechanisms.^35^, ^36^

Systemic inflammatory responses in patients with malignant tumours are considered to be an integral part of host response to tumours. The prognosis of cancer is not only affected by tumour‐related factors, but also by host‐related factors, especially systemic inflammatory responses, which are usually reflected by a variety of biochemical or haematological markers.^37^ In peripheral blood, neutrophils, lymphocytes and monocytes account for a large proportion of leukocytes, and lymphocytes are considered to play a fundamental role in cancer immune surveillance and mediation of cancer immunologic destruction.^38^ As an indicator of host inflammatory status, lymphocytes have been reported to contribute to the prediction of prognosis in various cancers, including renal cell carcinoma,^39^ breast cancer,^40^ and pancreatic cancer.^41^ Moreover, Suzuki et al. demonstrated that pretreatment total lymphocyte count was an important prognostic factor for patients with extensive‐stage SCLC.^42^ Zhang et al. reported that peripheral lymphocyte count could predict disease‐free survival (DFS) and overall survival (OS) in patients with NSCLC.^43^ However, the impact of pretreatment peripheral lymphocyte percentage in patients with lung cancer has not been elucidated yet.

To ensure the reliability of results, patients in this study were randomly assigned to either training or validation cohort, and the relationship between LY% and clinicopathological factors was investigated first. In addition to some slight inconsistencies regarding age and metastatic sites, the results of two cohorts illustrated that LY% was strongly correlated with other clinical parameters, as well as bone, liver and pleural metastasis in all lung cancer patients. Survival curves showed that low LY% had an obvious correlation with unfavourable survival status, and based on these findings, a multivariate cox regression analysis was performed to evaluate the prognostic value of LY%; it was then confirmed to be a significant predictor of prognosis in patients with lung cancer. In addition, the incorporation of LY% resulted in better predictive performance of clinical characteristic model on patient outcome. Neutrophil plays a complex role in inflammation within the tumour, and its elevated count has been demonstrated to have prognostic value in NSCLC.^44^ In this study, the role of NEUT% in lung cancer was examined as well. In spite of its relevance to clinical characteristics and survival outcomes, it could not make prediction for lung cancer prognosis in the multivariate analysis. Stratification analyses were also performed, and it was interesting to note that NEUT% could serve as prognostic factor in both SCLC and non‐metastatic patients. Regarding ADC and SCC subtypes, as well as adverse characteristics of advanced stage, undifferentiation and metastasis, LY% remained a more valuable predictor.

The current study shed light on peripheral lymphocyte percentage was significantly associated with prognosis of lung cancer patients. The percentage of lymphocyte has the potential to be a surrogate indicator of disease outcome and a stratification factor in clinical trials. While the results were derived based on the reference range of our clinical criteria, using the appropriate cut‐off value for the corresponding population is the best course of action. A ROC analysis might be useful to determine the optimal cut‐off level. Hence, further studies are required before it can be established as a validated prognostic marker. NLR is a well‐established predictor for patients with malignancy, which also is what mainly determines the LY%. In the subsequent analysis, the comparison for prognostic prediction potential of NLR and LY% will be conducted. In addition, we aim to figure out whether these two indicators can be combined as a more effective index to reflect the prognosis of patients with lung cancer. This study, conducted in a relatively large number of patients, obtained detailed clinical information to allow for extensive miscellaneous adjustments. However, data on the dynamic changes in lymphocyte and neutrophil percentages during tumour progression, and around the treatment period, were not available. Furthermore, the mechanism of complex association between inflammatory cells and tumour microenvironment has not been established, while the imbalance of lymphocyte and neutrophil ratio may provide insight into tumour progression and prognosis of individuals with lung cancer. We believe that the interaction or regulation of lymphocytes and neutrophils in lung cancer is worth studying and considering.

Evidence from the study supported the idea that pretreatment lymphocyte percentage effectively predicted the prognosis of lung cancer patients, and it was also an accurate response indicator in ADC and advanced lung cancer. Based on the results obtained, the integration of lymphocyte percentage with clinical characteristic model benefited the prognostic prediction of the disease.

## CONFLICT OF INTEREST

The authors confirm that there are no conflicts of interest.

## AUTHOR CONTRIBUTIONS

**Hong Huang:** Conceptualization (lead); Formal analysis (lead); Methodology (lead); Visualization (lead); Writing – original draft (lead); Writing – review & editing (lead). **Lei Li:** Data curation (equal); Investigation (lead); Resources (lead). **Wenxin Luo:** Data curation (equal); Investigation (lead); Resources (lead). **Yongfeng Yang:** Conceptualization (lead); Methodology (lead); Validation (lead); Writing – review & editing (equal). **Yinyun Ni:** Formal analysis (equal); Methodology (equal); Resources (equal); Validation (equal). **Tingting Song:** Formal analysis (equal); Methodology (equal); Software (equal); Visualization (equal). **Yihan Zhu:** Data curation (equal); Formal analysis (equal); Validation (equal). **Ying Yang:** Data curation (supporting); Formal analysis (equal); Investigation (supporting); Resources (supporting). **Li Zhang:** Conceptualization (lead); Funding acquisition (lead); Methodology (equal); Project administration (lead); Resources (lead); Supervision (lead); Validation (lead); Writing – original draft (equal); Writing – review & editing (lead).

## Supporting information

Table S1

Table S2

Table S3

Table S4

Table S5
