# Supplementary material for: Lymphocyte percentage as a valuable predictor of prognosis in lung cancer
Source: J Cell Mol Med. 2022 Feb 5;26(7):1918–31. doi: 10.1111/jcmm.17214 (PMC8980931; doi:10.1111/jcmm.17214)
Supplement: Supplementary file 1 — Table S1 [file JCMM-26-1918-s003.doc]

**Table S1. Demographics and clinical characteristics of enrolled lung cancer patients.**

| ***Characteristics*** | ***Training cohort (%)***  ***(n=667)*** | ***Validation cohort (%)***  ***(n=645)*** |
| --- | --- | --- |
| Sex  Male  Female  Age  <45 45–60 >60  Histological subtype  SCC  ADC  SCLC  Others  Stage  I  II  III  IV  Unknown  Smoking status  Never smoking  Current or ex-smoker  Differentiation  Undifferentiated  Poor  Moderate  Well  Unknown | 446(66.9)  221(33.1)  49(7.3)  286(42.9)  332(49.8)  167(25.0)  338(50.7)  119(17.8)  43(6.5)  57(8.5)  51(7.6)  170(25.5)  346(51.9)  43(6.5)  302(45.3)  365(54.7)  449(67.3)  91(13.7)  111(16.6)  4(0.6)  12(1.8) | 448(69.5)  197(30.5)  52(8.1)  291(45.1)  302(46.8)  171(26.5)  337(52.2)  94(14.6)  43(6.7)  55(8.5)  59(9.1)  160(24.8)  321(49.8)  50(7.8)  278(43.1)  367(56.9)  434(67.3)  92(14.3)  113(17.5)  4(0.6)  2(0.3) |
| Metastasis  No  Yes  Unknown  LY%  20-50  <20  NEUT%  40-75  >75 | 163(24.4)  467(70.0)  37(5.6)  365(54.7)  302(45.3)  484(72.6)  183(27.4) | 172(26.7)  427(66.3)  45(7.0)  362(56.1)  283(43.9)  461(71.5)  184(28.5) |

SCC: lung squamous carcinoma; ADC: lung adenocarcinoma; SCLC: small cell lung cancer;

LY%: lymphocyte percentage; NEUT%: neutrophil percentage; Poor: poorly differentiated; Moderate: moderately differentiated; Well: well differentiated
