# Supplementary material for: Lymphocyte percentage as a valuable predictor of prognosis in lung cancer
Source: J Cell Mol Med. 2022 Feb 5;26(7):1918–31. doi: 10.1111/jcmm.17214 (PMC8980931; doi:10.1111/jcmm.17214)
Supplement: Supplementary file 2 — Table S2 [file JCMM-26-1918-s005.docx]

**Table S2. The analysis of NEUT% in all lung cancer patients.**

| **Training cohort** | **No. (%)** | |  | |  |
| --- | --- | --- | --- | --- | --- |
|  | *40-75*  *(n=484)* | *>75*  *(n=183)* | *Total*  *(n=667)* | ***P value*** | |
| Basic Characteristics | | | | | |
| Age  <45  45-60  >60  Sex  Male  Female | 38(7.9)  215(44.4)  231(47.7)  320(66.1)  164(33.9) | 11(6.0)  71(38.8)  101(55.2)  126(68.9)  57(31.1) | 49  286  332  446  221 | 0.215  0.503 | |
| Histological subtype 0.000*** | | | | | |
| SCC  ADC  SCLC  Others  Stage  I  II  III  IV  Unknown  Smoking status  Never smoking  Current or ex-smoker  Differentiation  Undifferentiated  Poor  Moderate | 111(22.9)  239(49.4)  103(21.3)  31(6.4)  47(9.7)  41(8.5)  123(25.4)  240(49.6)  33(6.8)  217(44.8)  267(55.2)  320(66.1)  57(11.8)  95(19.6) | 58(31.7)  99(54.1)  16(8.7)  10(5.5)  10(5.5)  10(5.5)  47(25.7)  106(57.9)  10(5.5)  85(46.4)  98(53.6)  129(70.5)  34(18.6)  16(8.8) | 169  338  119  41  57  51  170  346  43  302  365  449  91  111 | 0.091  0.709  0.001** | |

| \| **Validation cohort** \| **No. (%)** \| \|  \| \|  \| \| --- \| --- \| --- \| --- \| --- \| --- \| \| *40-75*  *(n=461)* \| *>75*  *(n=184)* \| ***Total***  ***(n=645)*** \| ***P value*** \| \| | | | | |
| --- | --- | --- | --- | --- | --- | --- | --- | --- | --- | --- | --- | --- | --- | --- | --- |
| Basic Characteristics | | | | |
| Age  <45  45-60  >60  Sex  Male  Female  Histological subtype  SCC  ADC  SCLC  Others  Stage  I  II  III  IV  Unknown  Smoking status  Never smoking  Current or ex-smoker  Differentiation  Undifferentiated  Poor  Moderate | 39(8.4)  206(44.7)  216(46.9)  308(66.8)  153(33.2)  107(23.2)  259(56.2)  68(14.8)  27(5.8)  47(10.2)  45(9.8)  111(24.1)  220(47.7)  38(8.2)  205(44.5)  256(55.5)  302(65.5)  61(13.2)  94(20.4) | 13(7.1)  85(46.2)  86(46.7)  140(76.1)  44(23.9)  64(34.8)  78(42.4)  26(14.1)  16(8.7)  8(4.4)  14(7.6)  49(26.6)  101(54.9)  12(6.5)  73(39.7)  111(60.3)  132(71.7)  31(16.8)  19(10.3) | 52  291  302  448  197  171  337  94  43  55  59  160  321  50  278  367  434  92  113 | 0.825  0.021*  0.003**  0.040*  0.267  0.020* |

| Well  Unknown  Metastasis  Brain  No  Yes  Bone  No  Yes  Liver  No  Yes  Adrenal gland  No  Yes  Lymph node  No  Yes  Intrapulmonary  No  Yes  Pleural  No  Yes  Mediastinal  No  Yes | 3(0.6)  9(1.9)  438(90.5)  46(9.5)  407(84.1)  77(15.9)  448(92.6)  36(7.4)  455(94.0)  29(6.0)  214(44.2)  270(55.8)  427(88.2)  57(11.8)  431(89.0)  53(11.0)  472(97.5)  12(2.5) | 1(0.5)  3(1.6)  163(89.1)  20(10.9)  139(76.0)  44(24.0)  162(88.5)  21(11.5)  168(91.8)  15(8.2)  81(44.3)  102(55.7)  156(85.2)  27(14.8)  150(82.0)  33(18.0)  176(96.2)  7(3.8) | 4  12  601  66  546  121  610  57  623  44  295  372  583  84  581  86  648  19 | 0.582  0.015*  0.096  0.306  0.991  0.301  0.015*  0.351 |
| --- | --- | --- | --- | --- |

**P*<0.05, ***P*<0.01, ****P*<0.001. NEUT%: neutrophil percentage; SCC: lung squamous carcinoma; ADC: lung adenocarcinoma; SCLC: small cell lung cancer; Poor: poorly differentiated; Moderate: moderately differentiated; Well: well differentiated

| Well | 3(0.7) | 1(0.6) | 4 |  |
| --- | --- | --- | --- | --- |
| Unknown  Metastasis  Brain  No  Yes  Bone  No  Yes  Liver  No  Yes  Adrenal gland  No  Yes  Lymph node  No  Yes  Intrapulmonary  No  Yes  Pleural  No  Yes  Mediastinal  No  Yes | 1(0.2)  421(91.3)  40(8.7)  386(83.7)  75(16.3)  433(93.9)  28(6.1)  449(97.4)  12(2.6)  203(44.0)  258(56.0)  419(90.9)  42(9.1)  404(87.6)  57(12.4)  452(98.0)  9(2.0) | 1(0.6)  164(89.1)  20(10.9)  144(78.3)  40(21.7)  159(86.4)  25(13.6)  175(95.1)  9(4.9)  74(40.2)  110(59.8)  170(92.4)  14(7.6)  159(86.4)  25(13.6)  180(97.8)  4(2.2) | 2  585  60  530  115  592  53  624  21  277  368  589  56  563  82  632  13 | 0.387  0.101  0.002**  0.139  0.376  0.541  0.674  0.856 |
