# Supplementary material for: Lymphocyte percentage as a valuable predictor of prognosis in lung cancer
Source: J Cell Mol Med. 2022 Feb 5;26(7):1918–31. doi: 10.1111/jcmm.17214 (PMC8980931; doi:10.1111/jcmm.17214)
Supplement: Supplementary file 3 — Table S3 [file JCMM-26-1918-s002.docx]

**Table S3. The analysis of LY% in SCC and SCLC patients.**

| **SCC** | **No. (%)** | |  | |  |
| --- | --- | --- | --- | --- | --- |
|  | ***20-50***  ***(n=159)*** | ***<20***  ***(n=179)*** | ***Total***  ***(n=338)*** | ***P value*** | |
| Basic Characteristics | | | | | |
| Age  <45  45-60  >60  Sex  Male  Female | 6(3.8)  83(52.2)  70(44.0)  146(91.8)  13(8.2) | 4(2.3)  72(40.2)  103(57.5)  157(87.7)  22(12.3) | 10  155  173  303  35 | 0.043*  0.215 | |
| Stage  I  II  III  IV  Unknown  Smoking status  Never smoking  Current or ex-smoker  Differentiation  Undifferentiated  Poor  Moderate  Well  Unknown  Metastasis  Brain  No | 14(8.8)  23(14.5)  58(36.5)  57(35.8)  7(4.4)  30(18.9)  129(81.1)  81(50.9)  26(16.4)  47(29.6)  1(0.6)  4(2.5)  149(93.7) | 14(7.8)  17(9.5)  64(35.8)  76(42.5)  8(4.4)  35(19.6)  144(80.4)  104(58.1)  41(22.9)  28(15.7)  2(1.1)  4(2.2)  174(97.2) | 28  40  122  133  15  65  273  185  67  75  3  8  323 | 0.423  0.873  0.017*  0.119 | |

| \| **SCLC** \| **No. (%)** \| \|  \| \|  \| \| --- \| --- \| --- \| --- \| --- \| --- \| \| ***20-50***  ***(n=127)*** \| ***<20***  ***(n=86)*** \| ***Total***  ***(n=213)*** \| ***P value*** \| \| | | | | |
| --- | --- | --- | --- | --- | --- | --- | --- | --- | --- | --- | --- | --- | --- | --- | --- |
| Basic Characteristics | | | | |
| Age  <45  45-60  >60  Sex  Male  Female  Stage  I  II  III  IV  Unknown  Smoking status  Never smoking  Current or ex-smoker  Differentiation  Undifferentiated  Poor  Moderate  Well  Unknown  Metastasis  Brain  No | 10(7.9)  67(52.8)  50(39.4)  99(78.0)  28(22.0)  8(6.3)  9(7.1)  38(29.9)  49(38.6)  23(18.1)  39(30.7)  88(69.3)  108(85.0)  16(12.6)  1(0.8)  0(0.0)  2(1.6)  116(91.3) | 5(5.8)  40(46.5)  41(47.7)  73(84.9)  13(15.1)  2(2.3)  2(2.3)  22(25.6)  48(55.8)  12(14.0)  25(29.1)  61(70.9)  78(90.7)  8(9.3)  0(0.0)  0(0.0)  0(0.0)  77(89.5) | 15  107  91  172  41  10  11  60  97  35  64  149  186  24  1  0  2  193 | 0.464  0.208  0.048*  0.798  0.438  0.658 |

| Yes  Bone  No  Yes  Liver  No  Yes  Adrenal gland  No  Yes  Lymph node  No  Yes  Intrapulmonary  No  Yes  Pleural  No  Yes  Mediastinal  No  Yes | 10(6.3)  145(91.2)  14(8.8)  150(94.3)  9(5.7)  154(96.9)  5(3.1)  66(41.5)  93(58.5)  144(90.6)  15(9.4)  149(93.7)  10(6.3)  157(98.7)  2(1.3) | 5(2.8)  152(84.9)  27(15.1)  163(91.1)  16(8.9)  171(95.5)  8(4.5)  79(44.1)  100(55.9)  162(90.5)  17(9.5)  160(89.4)  19(10.6)  172(96.1)  7(3.9) | 15  297  41  313  25  325  13  145  193  306  32  309  29  329  9 | 0.078  0.250  0.527  0.627  0.984  0.156  0.131 |
| --- | --- | --- | --- | --- |

**P*<0.05. LY%: lymphocyte percentage; SCC: lung squamous carcinoma; SCLC: small cell lung cancer; Poor: poorly differentiated; Moderate: moderately differentiated; Well: well differentiated

| Yes  Bone  No  Yes  Liver  No  Yes  Adrenal gland  No  Yes  Lymph node  No  Yes  Intrapulmonary  No  Yes  Pleural  No  Yes  Mediastinal  No  Yes | 11(8.7)  116(91.3)  11(8.7)  116(91.3)  11(8.7)  117(92.1)  10(7.9)  50(39.4)  77(60.6)  121(95.3)  6(4.7)  123(96.9)  4(3.1)  122(96.1)  5(3.9) | 9(10.5)  72(83.7)  14(16.3)  70(81.4)  16(18.6)  78(90.7)  8(9.3)  20(23.3)  66(76.7)  78(90.7)  8(9.3)  76(88.4)  10(11.6)  84(97.7)  2(2.3) | 20  188  25  186  27  195  18  70  143  199  14  199  14  206  7 | 0.090  0.032*  0.713  0.014*  0.186  0.014*  0.517 |
| --- | --- | --- | --- | --- |
