# Supplementary material for: Lymphocyte percentage as a valuable predictor of prognosis in lung cancer
Source: J Cell Mol Med. 2022 Feb 5;26(7):1918–31. doi: 10.1111/jcmm.17214 (PMC8980931; doi:10.1111/jcmm.17214)
Supplement: Supplementary file 4 — Table S4 [file JCMM-26-1918-s001.docx]

**Table S4. Classification analyses of NEUT% in ADC, SCC, and SCLC.**

| **ADC** | **No. (%)** |  |  |  |
| --- | --- | --- | --- | --- |
|  | ***40-75***  ***(n=498)*** | ***>75***  ***(n=177)*** | ***Total***  ***(n=675)*** | ***P value*** |
| Basic Characteristics | | | | |
| Age  <45  45-60  >60  Sex  Male  Female | 50(10.0)  203(40.8)  245(49.2)  256(51.4)  242(48.6) | 17(9.6)  76(42.9)  84(47.5)  105(59.3)  72(40.7) | 67  279  329  361  314 | 0.880  0.070 |
| Stage  I  II  III  IV  Unknown  Smoking status  Never smoking  Current or ex-smoker  Differentiation  Undifferentiated  Poor  Moderate  Well  Unknown  Metastasis  Brain  No  Yes  Bone  No  Yes  Liver  No  Yes  Adrenal gland  No  Yes  Lymph node  No | 60(12.0)  42(8.4)  84(16.9)  289(58.0)  23(4.7)  305(61.2)  193(38.8)  313(62.9)  49(9.8)  127(25.5)  4(0.8)  5(1.0)  441(88.6)  57(11.4)  391(78.5)  107(21.5)  467(93.8)  31(6.2)  484(97.2)  14(2.8)  231(46.4) | 7(4.0)  9(5.1)  39(22.0)  111(62.7)  11(6.2)  105(59.3)  72(40.7)  132(74.6)  25(14.1)  19(10.7)  1(0.6)  0(0.0)  149(84.2)  28(15.8)  128(72.3)  49(27.7)  156(88.1)  21(11.9)  162(91.5)  15(8.5)  75(42.4) | 67  51  123  400  34  410  265  445  74  146  5  5  590  85  519  156  623  52  646  29  306 | 0.005**  0.653  0.000***  0.132  0.093  0.016*  0.001**  0.357 |
| Yes  Intrapulmonary  No  Yes  Pleural  No  Yes  Mediastinal  No  Yes | 267(53.6)  436(87.6)  62(12.4)  418(83.9)  80(16.1)  490(98.4)  8(1.6) | 102(57.6)  155(87.6)  22(12.4)  138(78.0)  39(22.0)  170(96.0)  7(4.0) | 369  591  84  556  119  660  15 | 0.994  0.073  0.069 |

**P*<0.05, ***P*<0.01, ****P*<0.001. NEUT%: neutrophil percentage; ADC: lung adenocarcinoma; Poor: poorly differentiated; Moderate: moderately differentiated; Well: well differentiated

| **SCC** | **No. (%)** |  |  |  |
| --- | --- | --- | --- | --- |
|  | ***40-75***  ***(n=218)*** | ***>75***  ***(n=120)*** | ***Total***  ***(n=338)*** | ***P value*** |
| Basic Characteristics | | | | |
| Age  <45  45-60  >60  Sex  Male  Female | 7(3.2)  104(47.7)  107(49.1)  196(89.9)  22(10.1) | 3(2.5)  51(42.5)  66(55.0)  107(89.2)  13(10.8) | 10  155  173  303  35 | 0.571  0.830 |
| Stage  I  II  III  IV  Unknown  Smoking status  Never smoking  Current or ex-smoker  Differentiation  Undifferentiated  Poor  Moderate  Well  Unknown  Metastasis  Brain  No  Yes  Bone  No  Yes  Liver  No  Yes  Adrenal gland  No  Yes  Lymph node  No  Yes  Intrapulmonary  No  Yes  Pleural  No  Yes  Mediastinal  No  Yes | 18(8.3)  28(12.8)  86(39.4)  78(35.8)  8(3.7)  41(18.8)  177(81.2)  114(52.3)  38(17.4)  60(27.5)  2(0.9)  4(1.9)  207(95.0)  11(5.0)  199(91.3)  19(8.7)  206(94.5)  12(5.5)  209(95.9)  9(4.1)  89(40.8)  129(59.2)  200(91.7)  18(8.3)  201(92.2)  17(7.8)  212(97.2)  6(2.8) | 10(8.4)  12(10.0)  36(30.0)  55(45.8)  7(5.8)  24(20.0)  96(80.0)  71(59.2)  29(24.2)  15(12.5)  1(0.8)  4(3.3)  116(96.7)  4(3.3)  98(81.7)  22(18.3)  107(89.2)  13(10.8)  116(96.7)  4(3.3)  56(46.7)  64(53.3)  106(88.3)  14(11.7)  108(90.0)  12(10.0)  117(97.5)  3(2.5) | 28  40  122  133  15  65  273  185  67  75  3  8  323  15  297  41  313  25  325  13  145  193  306  32  309  29  329  9 | 0.219  0.790  0.011*  0.464  0.010*  0.073  0.716  0.299  0.306  0.489  0.890 |

**P*<0.05. NEUT%: neutrophil percentage; SCC: lung squamous carcinoma; Poor: poorly differentiated; Moderate: moderately differentiated; Well: well differentiated

| **SCLC** | **No. (%)** |  |  |  |
| --- | --- | --- | --- | --- |
|  | ***40-75***  ***(n=171)*** | ***>75***  ***(n=42)*** | ***Total***  ***(n=213)*** | ***P value*** |
| Basic Characteristics | | | | |
| Age  <45  45-60  >60  Sex  Male  Female | 14(8.2)  89(52.0)  68(39.8)  138(80.7)  33(19.3) | 1(2.4)  18(42.8)  23(54.8)  34(81.0)  8(19.0) | 15  107  91  172  41 | 0.138  0.971 |
| Stage  I  II  III  IV  Unknown  Smoking status  Never smoking  Current or ex-smoker  Differentiation  Undifferentiated  Poor  Moderate  Well  Unknown  Metastasis  Brain  No  Yes  Bone  No  Yes  Liver  No  Yes  Adrenal gland  No  Yes  Lymph node  No  Yes  Intrapulmonary  No  Yes  Pleural  No  Yes  Mediastinal  No  Yes | 10(5.8)  10(5.8)  49(28.8)  70(40.9)  32(18.7)  50(29.2)  121(70.8)  150(87.7)  18(10.5)  1(0.6)  0(0.0)  2(1.2)  156(91.2)  15(8.8)  154(90.1)  17(9.9)  154(90.1)  17(9.9)  157(91.8)  14(8.2)  61(35.7)  110(64.3)  160(93.6)  11(6.4)  162(94.7)  9(5.3)  165(96.5)  6(3.5) | 0(0.0)  1(2.4)  11(26.2)  27(64.3)  3(7.1)  14(33.3)  28(66.7)  36(85.7)  6(14.3)  0(0.0)  0(0.0)  0(0.0)  37(88.1)  5(11.9)  34(81.0)  8(19.0)  32(76.2)  10(23.8)  38(90.5)  4(9.5)  9(21.4)  33(78.6)  39(92.9)  3(7.1)  37(88.1)  5(11.9)  41(97.6)  1(2.4) | 10  11  60  97  35  64  149  186  24  1  0  2  193  20  188  25  186  27  195  18  70  143  199  14  199  14  206  7 | 0.039*  0.604  0.566  0.533  0.100  0.016*  0.780  0.078  0.868  0.120  0.713 |

**P*<0.05. NEUT%: neutrophil percentage; SCLC: small cell lung cancer; Poor: poorly differentiated; Moderate: moderately differentiated; Well: well differentiated
