# Supplementary material for: Lymphocyte percentage as a valuable predictor of prognosis in lung cancer
Source: J Cell Mol Med. 2022 Feb 5;26(7):1918–31. doi: 10.1111/jcmm.17214 (PMC8980931; doi:10.1111/jcmm.17214)
Supplement: Supplementary file 5 — Table S5 [file JCMM-26-1918-s004.docx]

**Table S5. Univariate and multivariate survival analysis in SCC patients.**

| **Variables** | **Univariate analysis**  **HR (95% CI)** | **P value** | **Multivariate**  **analysis**  **HR (95% CI)** | **P value** |
| --- | --- | --- | --- | --- |
| Age  <45 45–60 >60  Sex  Male  Female | 1[Reference]  1.547(0.567-4.221)  3.312(1.220-8.996)  1[Reference]  0.948(0.598-1.502) | 0.394  0.019*  0.820 | 1[Reference]  1.468(0.533-4.048)  3.183(1.159-8.741) | 0.458  0.025* |
| Stage  I  II  III  IV  Smoking status  Never smoking  Current or ex-smoker  Differentiation  Undifferentiated  Poor  Moderate  Well | 1[Reference]  0.906(0.464-1.771)  1.638(0.943-2.846)  1.718(0.993-2.972)  1[Reference]  0.847(0.608-1.180)  1[Reference]  0.499(0.341-0.729)  0.472(0.328-0.679)  0.589(0.146-2.382) | 0.774  0.080  0.053  0.326  0.000***  0.000***  0.458 | 1[Reference]  0.597(0.403-0.885)  0.558(0.376-0.828)  2.151(0.520-8.893) | 0.010*  0.004**  0.290 |
| Metastasis  No  Yes  NEUT%  40-75  >75  LY%  20-50  <20 | 1[Reference]  1.743(1.263-2.405)  1[Reference]  1.407(1.070-1.851)  1[Reference]  1.420(1.085-1.859) | 0.001**  0.015*  0.011* | 1[Reference]  1.768(1.242-2.516)  1[Reference]  1.424(1.060-1.913) | 0.002**  0.019* |

**P*<0.05, ***P*<0.01, ****P*<0.001. SCC: lung squamous carcinoma; NEUT%: neutrophil percentage; LY%: lymphocyte percentage; Poor: poorly differentiated; Moderate: moderately differentiated; Well: well differentiated

**Univariate and multivariate survival analysis in SCLC patients.**

| **Univariate**  **analysis**  **HR (95% CI)** | **P value** | **Multivariate**  **analysis**  **HR (95% CI)** | **P value** |
| --- | --- | --- | --- |
| 1[Reference]  0.705(0.361-1.377)  1.099(0.563-2.145)  1[Reference]  0.651(0.415-1.021) | 0.306  0.783  0.061 |  |  |
| 1[Reference]  1.733(0.489-6.143)  1.678(0.596-4.723)  2.658(0.970-7.282)  1[Reference]  1.411(0.969-2.055)  1[Reference]  1.314(0.767-2.251) | 0.395  0.327  0.057  0.072  0.320  0.961 |  |  |
| 1[Reference]  1.706(1.103-2.637)  1[Reference]  1.818(1.238-2.670)  1[Reference]  1.719(1.227-2.409) | 0.016*  0.002**  0.002** | 1[Reference]  1.794(1.069-3.012)  1[Reference]  1.592(1.054-2.407) | 0.027*  0.027* |

SCLC: small cell lung cancer

**Univariate and multivariate survival analysis in stage III patients.**

| **Variables** | **Univariate analysis**  **HR (95% CI)** | **P value** | **Multivariate**  **analysis**  **HR (95% CI)** | **P value** |
| --- | --- | --- | --- | --- |
| Age  <45 45–60 >60  Sex  Male  Female | 1[Reference]  0.614(0.280-1.346)  1.142(0.534-2.442)  1[Reference]  0.657(0.450-0.958) | 0.223  0.732  0.029* |  |  |
| Histological subtype  Others  SCC  ADC  SCLC  Smoking status  Never smoking  Current or ex-smoker  Differentiation  Undifferentiated  Poor  Moderate  Well | 1[Reference]  1.313(0.730-2.361)  1.222(0.677-2.206)  1.086(0.574-2.055)  1[Reference]  1.289(0.951-1.745)  1[Reference]  0.655(0.424-1.010)  0.636(0.437-0.924)  0.642(0.090-4.597) | 0.363  0.505  0.799  0.101  0.055  0.018*  0.659 |  |  |
| Metastasis  No  Yes  NEUT%  40-75  >75  LY%  20-50  <20 | 1[Reference]  1.458(1.003-2.120)  1[Reference]  1.548(1.157-2.073)  1[Reference]  1.698(1.287-2.241) | 0.048*  0.003**  0.000*** | 1[Reference]  1.476(1.005-2.167)  1[Reference]  1.698(1.263-2.284) | 0.047*  0.000*** |

**P*<0.05, ***P*<0.01, ****P*<0.001. SCC: lung squamous carcinoma; ADC: lung adenocarcinoma; SCLC: small cell lung cancer; NEUT%: neutrophil percentage; LY%: lymphocyte percentage; Poor: poorly differentiated; Moderate: moderately differentiated; Well: well differentiated

**Univariate and multivariate survival analysis in stage IV patients.**

| **Univariate**  **analysis**  **HR (95% CI)** | **P value** | **Multivariate**  **analysis**  **HR (95% CI)** | **P value** |
| --- | --- | --- | --- |
| 1[Reference]  0.977(0.721-1.325)  1.474(1.091-1.992) | 0.883  0.011* | 1[Reference]  1.038(0.755-1.428)  1.485(1.083-2.036) | 0.818  0.014* |
| 1[Reference]  0.952(0.789-1.149)  1[Reference]  0.813(0.528-1.251)  0.908(0.612-1.349)  0.995(0.640-1.547)  1[Reference]  1.193(0.995-1.431)  1[Reference]  1.007(0.778-1.304)  0.365(0.224-0.594)  0.429(0.107-1.724)  1[Reference]  1.882(1.359-2.606)  1[Reference]  1.608(1.329-1.945)  1[Reference]  1.666(1.387-2.001) | 0.606  0.346  0.634  0.982  0.057  0.957  0.000***  0.233  0.000***  0.000***  0.000*** | 1[Reference]  0.995(0.765-1.294)  0.454(0.277-0.744)  0.955(0.235-3.879)  1[Reference]  1.549(1.110-2.162)  1[Reference]  1.575(1.303-1.903) | 0.968  0.002**  0.949  0.010*  0.000*** |

**Univariate and multivariate survival analysis in undifferentiated patients.**

| **Variables** | **Univariate analysis**  **HR (95% CI)** | **P value** | **Multivariate**  **analysis**  **HR (95% CI)** | **P value** |
| --- | --- | --- | --- | --- |
| Age  <45 45–60 >60  Sex  Male  Female | 1[Reference]  0.858(0.630-1.170)  1.295(0.956-1.755)  1[Reference]  0.923(0.779-1.094) | 0.333  0.095  0.357 | 1[Reference]  0.981(0.701-1.374)  1.460(1.049-2.033) | 0.913  0.025* |
| Histological subtype  Others  SCC  ADC  SCLC  Smoking status  Never smoking  Current or ex-smoker  Stage  I  II  III  IV | 1[Reference]  1.080(0.772-1.511)  1.058(0.773-1.446)  0.859(0.610-1.209)  1[Reference]  1.146(0.977-1.344)  1[Reference]  2.131(1.130-4.018)  1.893(1.073-3.342)  2.231(1.283-3.879) | 0.654  0.726  0.383  0.094  0.019*  0.028*  0.004** | 1[Reference]  1.932(0.966-3.864)  1.479(0.779-2.810)  1.868(0.992-3.519) | 0.063  0.232  0.053 |
| Metastasis  No  Yes  NEUT%  40-75  >75  LY%  20-50  <20 | 1[Reference]  1.419(1.149-1.753)  1[Reference]  1.627(1.376-1.924)  1[Reference]  1.703(1.453-1.998) | 0.001**  0.000***  0.000*** | 1[Reference]  1.278(0.991-1.647)  1[Reference]  1.655(1.390-1.969) | 0.058  0.000*** |

**P*<0.05, ***P*<0.01, ****P*<0.001. SCC: lung squamous carcinoma; ADC: lung adenocarcinoma; SCLC: small cell lung cancer; NEUT%: neutrophil percentage; LY%: lymphocyte percentage;

**Univariate and multivariate survival analysis in differentiated patients.**

| **Univariate**  **analysis**  **HR (95% CI)** | **P value** | **Multivariate**  **analysis**  **HR (95% CI)** | **P value** |
| --- | --- | --- | --- |
| 1[Reference]  0.666(0.497-0.892)  0.981(0.737-1.308)  1[Reference]  0.812(0.681-0.969)  1[Reference] | 0.006**  0.898  0.021* | 1[Reference]  0.788(0.579-1.073)  1.193(0.881-1.616) | 0.130  0.254 |
| 0.656(0.472-0.913) | 0.012* |  |  |
| 0.635(0.462-0.872)  1.088(0.762-1.552)  1[Reference]  1.257(1.070-1.478)  1[Reference]  1.337(0.828-2.159)  3.242(2.194-4.791)  4.453(3.063-6.475)  1[Reference]  2.485(2.025-3.049)  1[Reference]  1.489(1.260-1.759)  1[Reference]  1.630(1.392-1.909) | 0.005**  0.643  0.006**  0.234  0.000***  0.000***  0.000***  0.000***  0.000*** | 1[Reference]  1.253(1.048-1.499)  1[Reference]  1.171(0.713-1.922)  2.203(1.440-3.370)  3.228(2.139-4.870)  1[Reference]  1.674(1.308-2.141)  1[Reference]  1.358(1.144-1.613) | 0.014*  0.533  0.000***  0.000***  0.000***  0.000*** |

**Univariate and multivariate survival analysis in patients without metastasis.**

| **Variables** | **Univariate analysis**  **HR (95% CI)** | **P value** | **Multivariate**  **analysis**  **HR (95% CI)** | **P value** |
| --- | --- | --- | --- | --- |
| Age  <45 45–60 >60  Sex  Male  Female | 1[Reference]  0.538(0.286-1.014)  0.554(0.290-1.058)  1[Reference]  0.863(0.612-1.215) | 0.055  0.073  0.398 |  |  |
| Histological subtype  Others  SCC  ADC  SCLC  Smoking status  Never smoking  Current or ex-smoker  Stage  I  II  III  IV  Differentiation  Undifferentiated  Poor  Moderate  Well | 1[Reference]  0.623(0.354-1.098)  0.427(0.245-0.743)  0.685(0.365-1.286)  1[Reference]  1.285(0.928-1.779)  1[Reference]  1.529(0.880-2.654)  2.070(1.219-3.515)  2.133(1.277-3.566)  1[Reference]  0.330(0.177-0.615)  0.284(0.187-0.431)  0.449(0.111-1.820) | 0.102  0.003**  0.239  0.130  0.132  0.007**  0.004**  0.000***  0.000***  0.262 | 1[Reference]  0.327(0.169-0.630)  0.307(0.197-0.479)  0.451(0.111-1.837) | 0.001**  0.000***  0.267 |
| NEUT%  40-75  >75  LY%  20-50  <20 | 1[Reference]  1.948(1.379-2.752)  1[Reference]  1.963(1.423-2.709) | 0.000***  0.000*** | 1[Reference]  1.958(1.329-2.883) | 0.001** |

**Univariate and multivariate survival analysis in patients with metastasis.**

| **Univariate**  **analysis**  **HR (95% CI)** | **P value** | **Multivariate**  **analysis**  **HR (95% CI)** | **P value** |
| --- | --- | --- | --- |
| 1[Reference]  0.967(0.709-1.320)  1.548(1.142-2.098)  1[Reference]  0.862(0.722-1.029)  1[Reference]  1.119(0.760-1.647)  1.158(0.800-1.676)  1.176(0.789-1.754)  1[Reference]  1.239(1.052-1.458)  1[Reference]  1.789(0.747-4.283)  3.172(1.486-6.772)  4.103(1.943-8.665)  1[Reference]  0.917(0.733-1.148)  0.443(0.329-0.597)  0.370(0.052-2.632)  1[Reference]  1.436(1.209-1.705)  1[Reference]  1.611(1.371-1.892) | 0.834  0.005**  0.100  0.570  0.436  0.425  0.010*  0.192  0.003**  0.000***  0.450  0.000***  0.320  0.000***  0.000*** | 1[Reference]  0.998(0.725-1.374)  1.624(1.187-2.221)  1[Reference]  1.196(1.008-1.419)  1[Reference]  1.223(0.503-2.969)  1.901(0.870-4.154)  2.481(1.140-5.397)  1[Reference]  0.947(0.753-1.191)  0.584(0.424-0.804)  0.614(0.084-4.467)  1[Reference]  1.529(1.294-1.806) | 0.990  0.002**  0.040*  0.657  0.107  0.022*  0.640  0.001**  0.630  0.000*** |

**P*<0.05, ***P*<0.01, ****P*<0.001. SCC: lung squamous carcinoma; ADC: lung adenocarcinoma; SCLC: small cell lung cancer; NEUT%: neutrophil percentage; LY%: lymphocyte percentage; Poor: poorly differentiated; Moderate: moderately differentiated; Well: well differentiated
